# Supplementary material for: Chronic intermittent hypoxia aggravated diabetic cardiomyopathy through LKB1/AMPK/Nrf2 signaling pathway
Source: PLoS One. 2024 Mar 7;19(3):e0296792. doi: 10.1371/journal.pone.0296792 (PMC10919874; doi:10.1371/journal.pone.0296792)
Supplement: S1 Graphical abstract — (PDF) [file pone.0296792.s001.pdf]

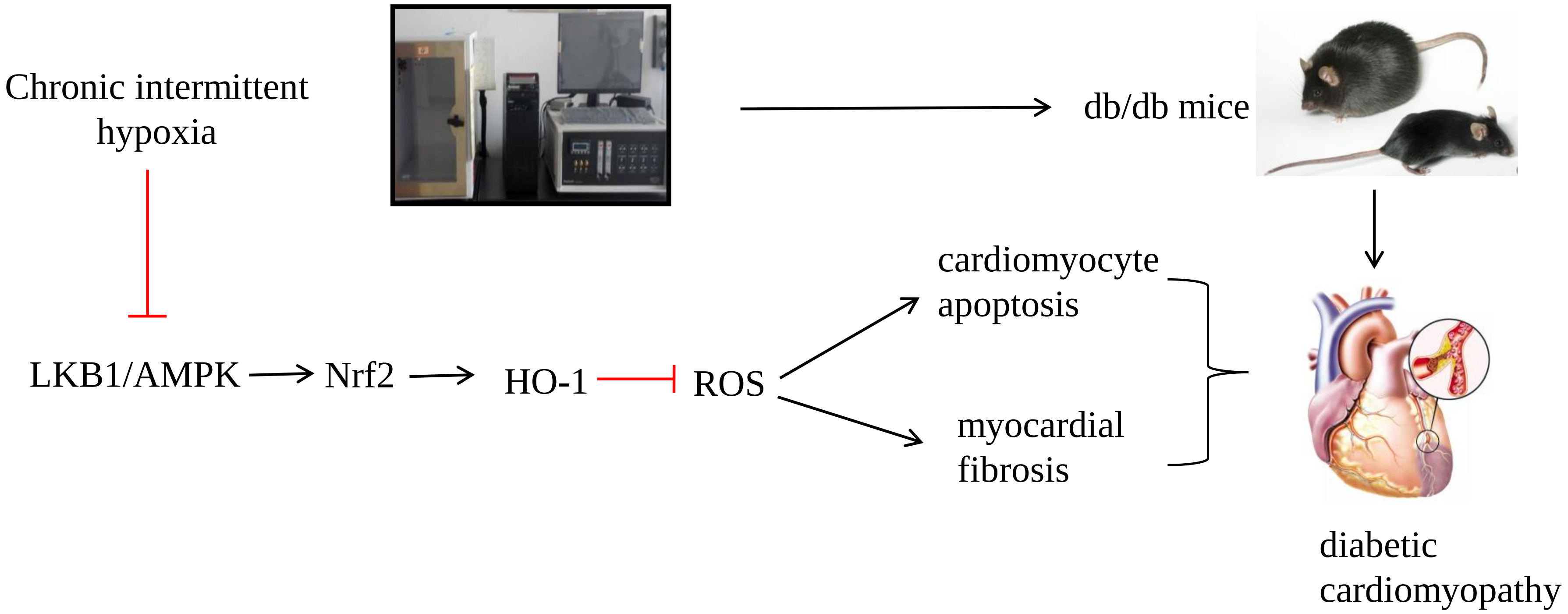
Chronic intermittent hypoxia
db/db mice
cardiomyocyte apoptosis
LKB1/AMPK
Nrf2
HO-1
ROS
myocardial fibrosis
diabetic cardiomyopathy
