# Supplementary material for: Chronic intermittent hypoxia aggravated diabetic cardiomyopathy through LKB1/AMPK/Nrf2 signaling pathway
Source: PLoS One. 2024 Mar 7;19(3):e0296792. doi: 10.1371/journal.pone.0296792 (PMC10919874; doi:10.1371/journal.pone.0296792)

Supplementary Figures

Chronic intermittent hypoxia aggravated diabetic cardiomyopathy through LKB1/AMPK/Nrf2 signaling pathway

Bingbing Liu[a,#], Jianchao Si[a,#], Kerong Qi[a#],Dongli Li[a], Tingting Li[a], Yi Tang[a], Shengchang Yang[a,b]*, Ensheng Ji[a,b]*

Figure 2: Representative blot images of PI3K, AKT, GLUT4 expression.

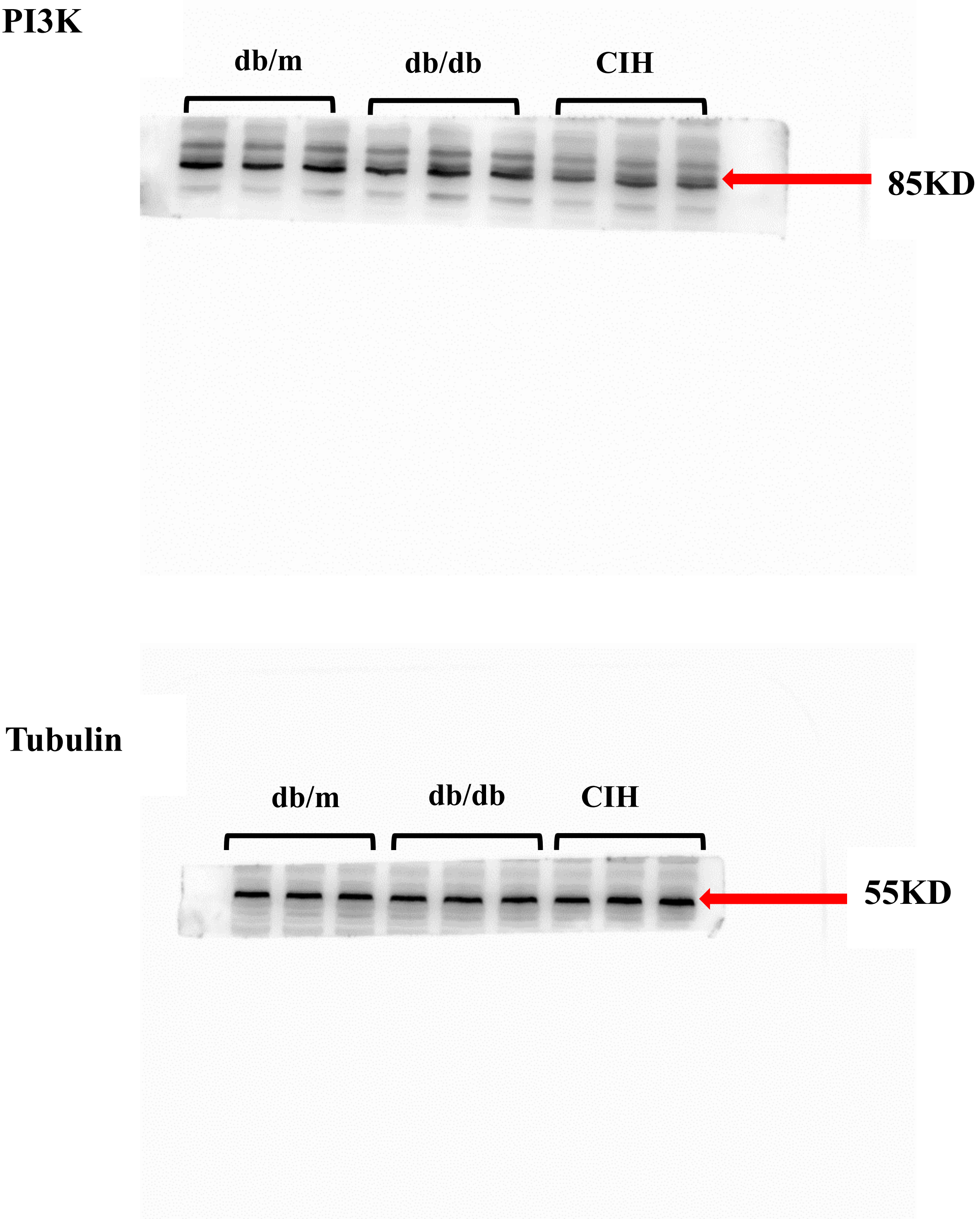

P-AKT

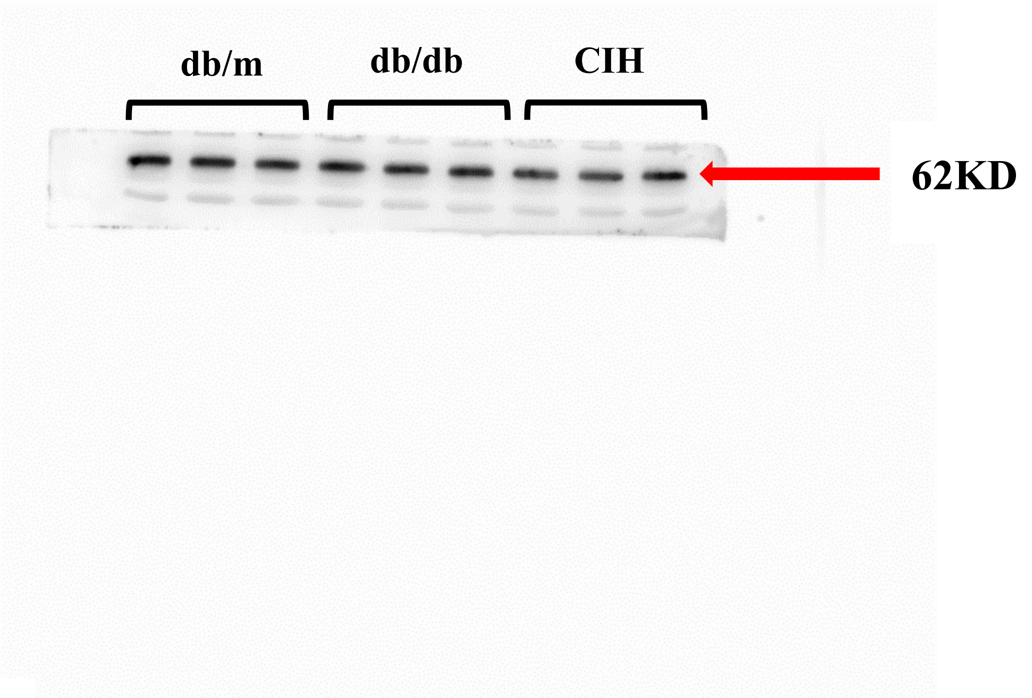

P-AKT

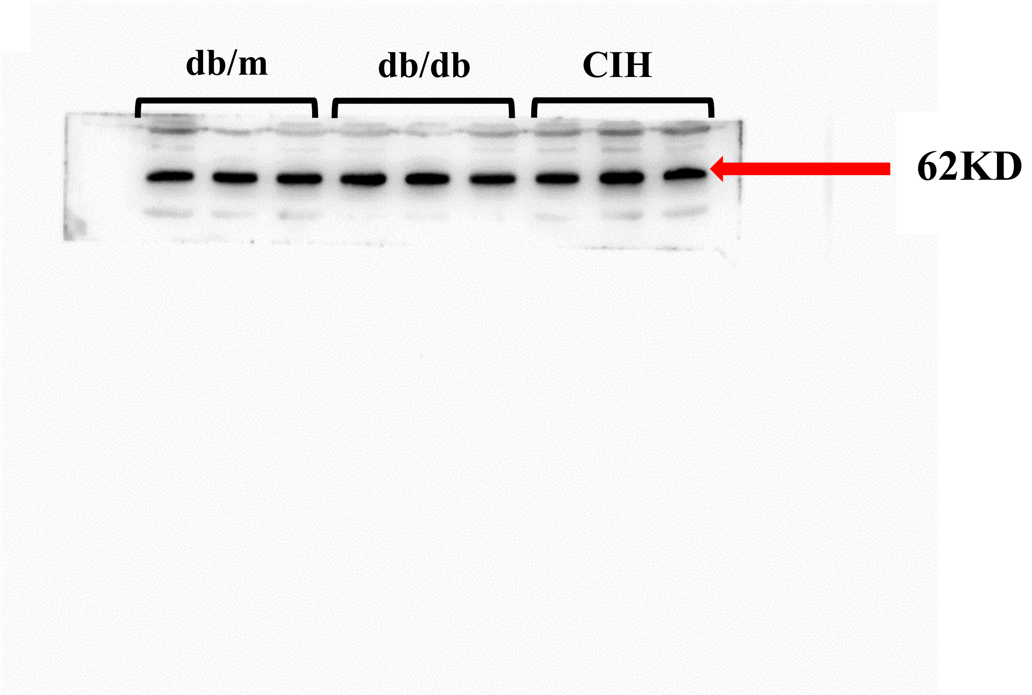

GLUT4

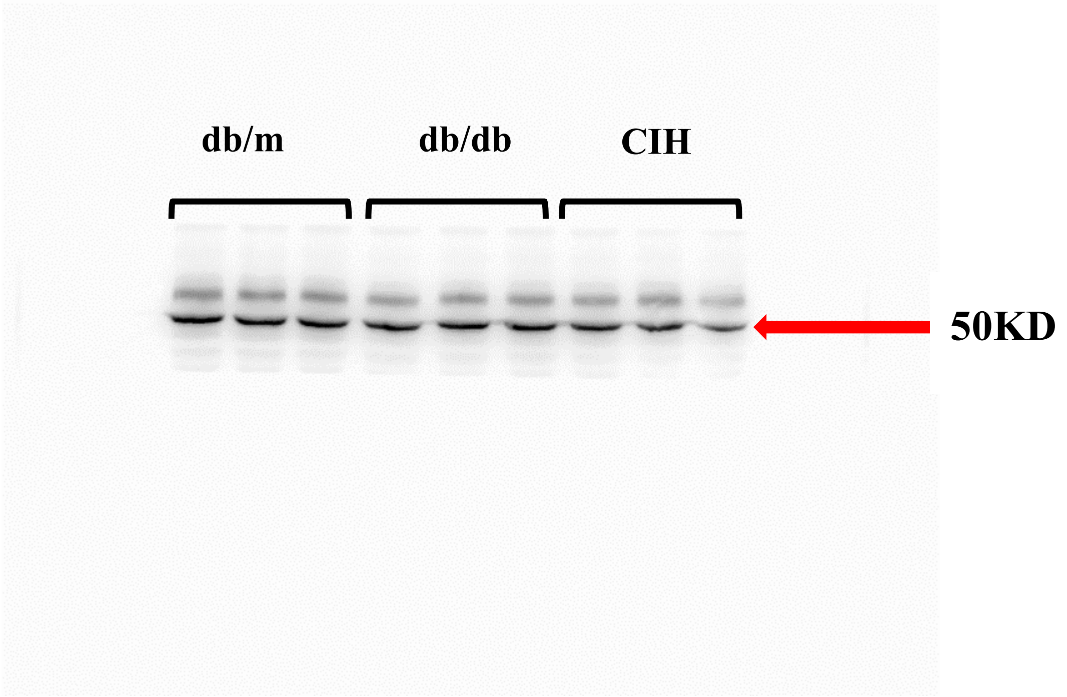

Tubulin

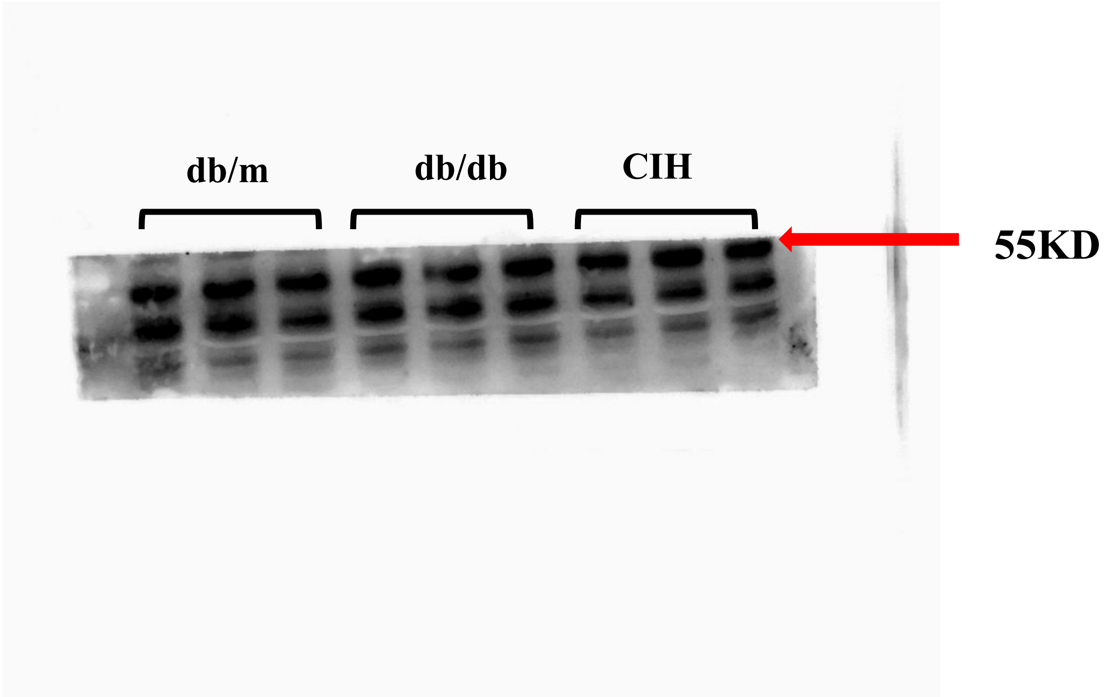

Figure 5: Representative blot images of Bax, Bcl-2, Caspase-3 expression.

Bax

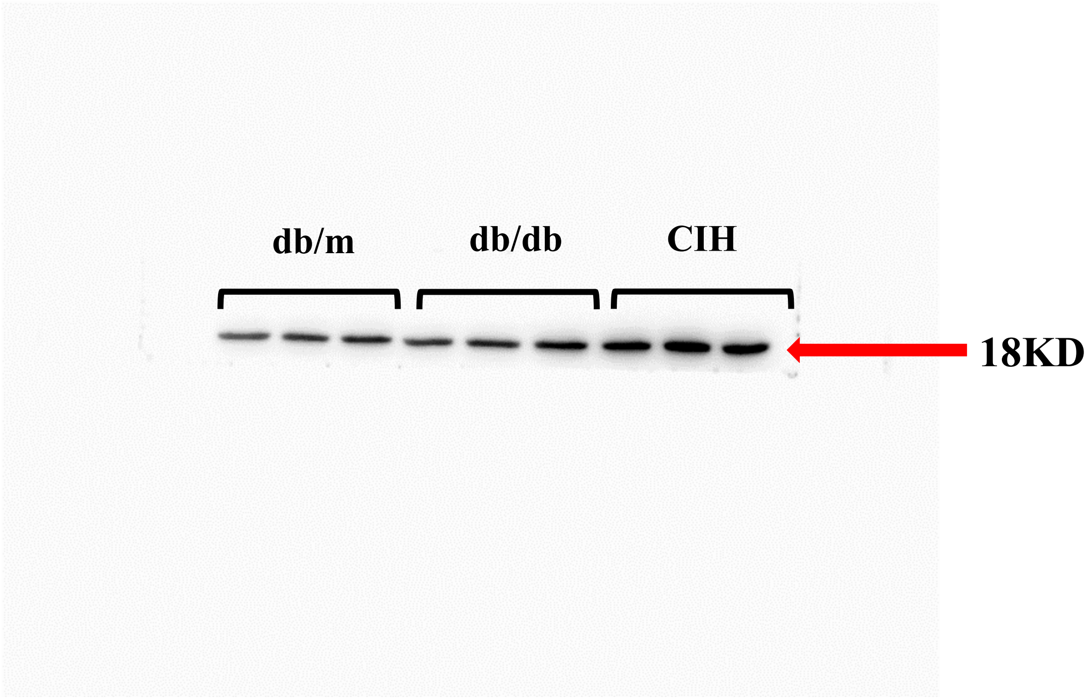

Bcl-2

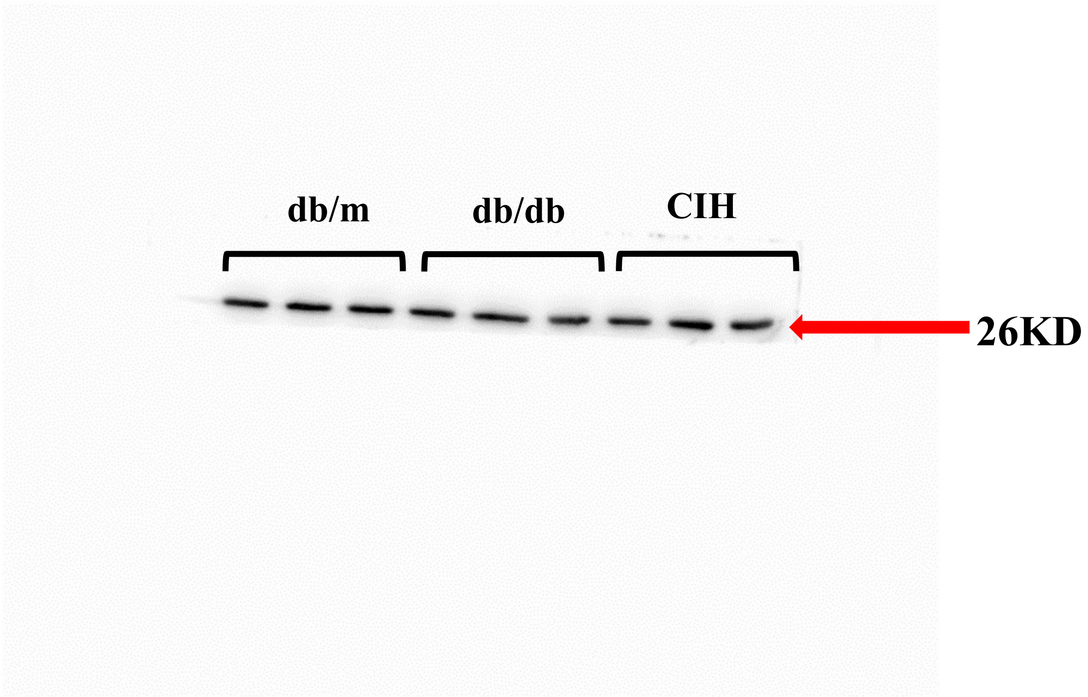

Caspase-3

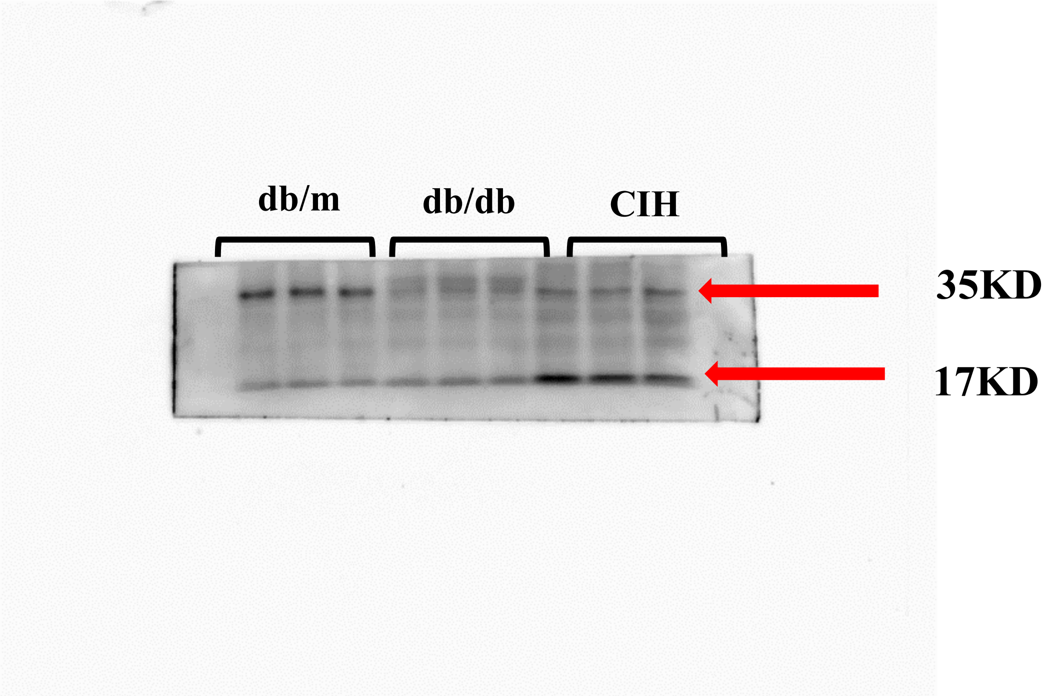

Figure 7: Representative blot images of LKB1, AMPK, Nrf2, and HO-1 expression.

P-LKB1

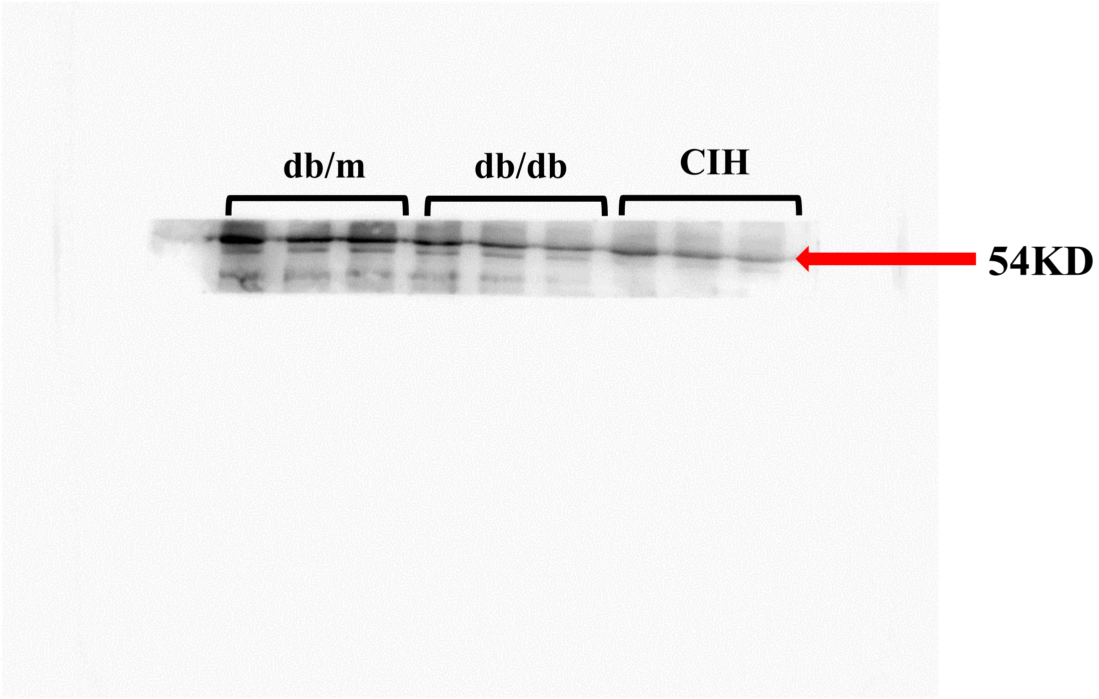

LKB1

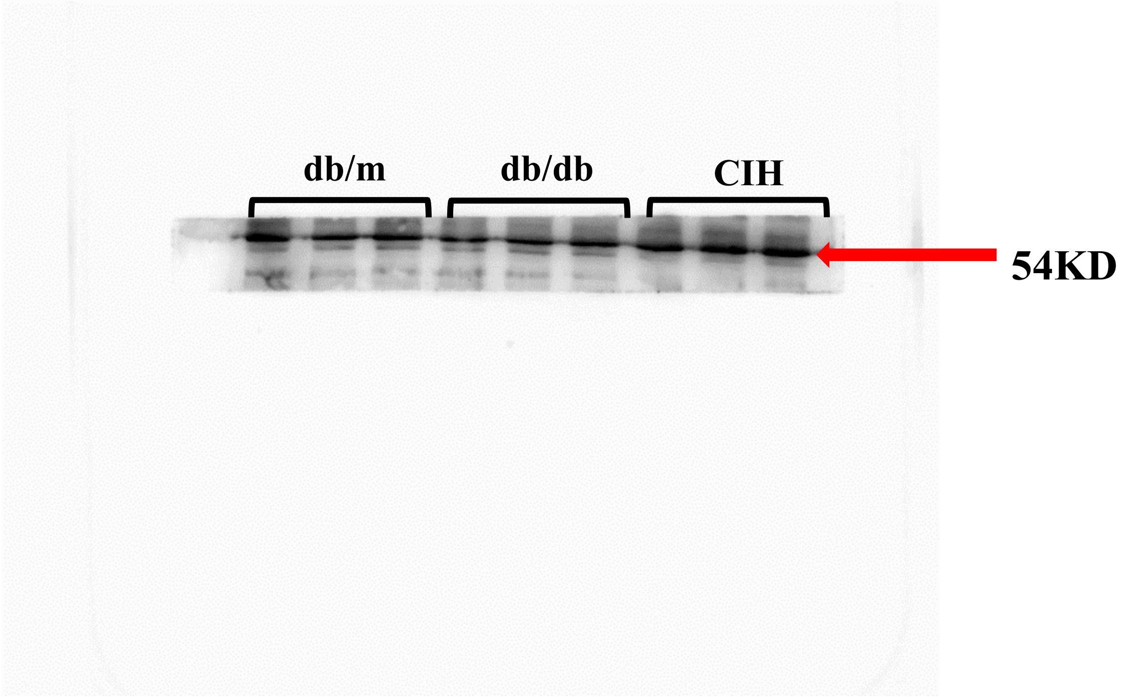

P-AMPK

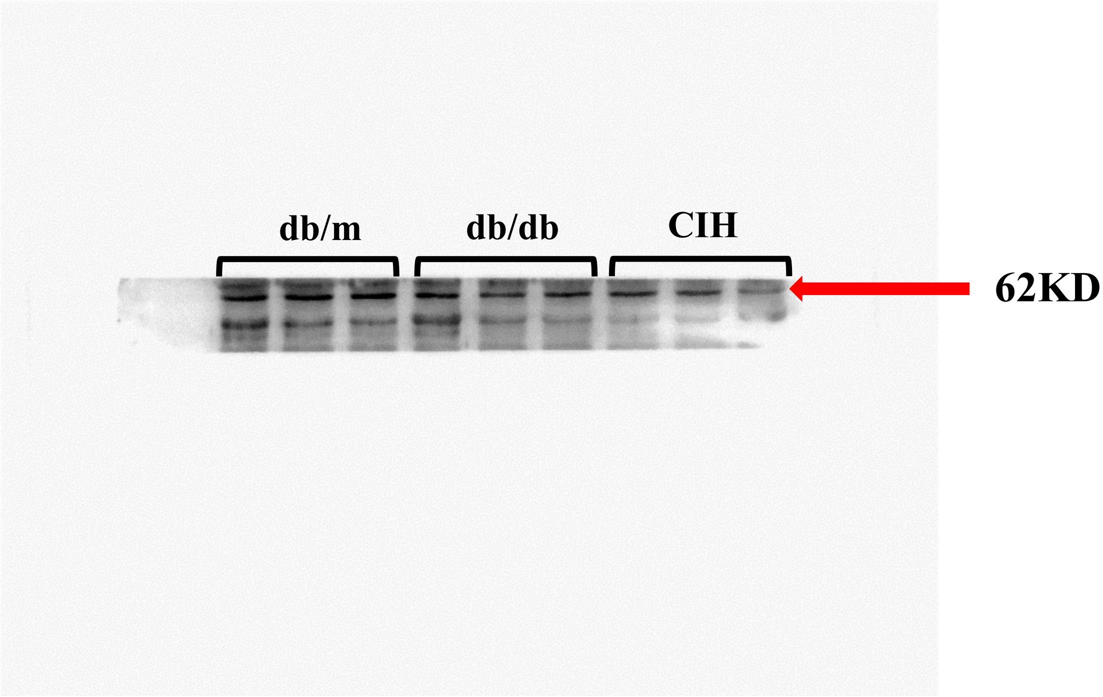

AMPK

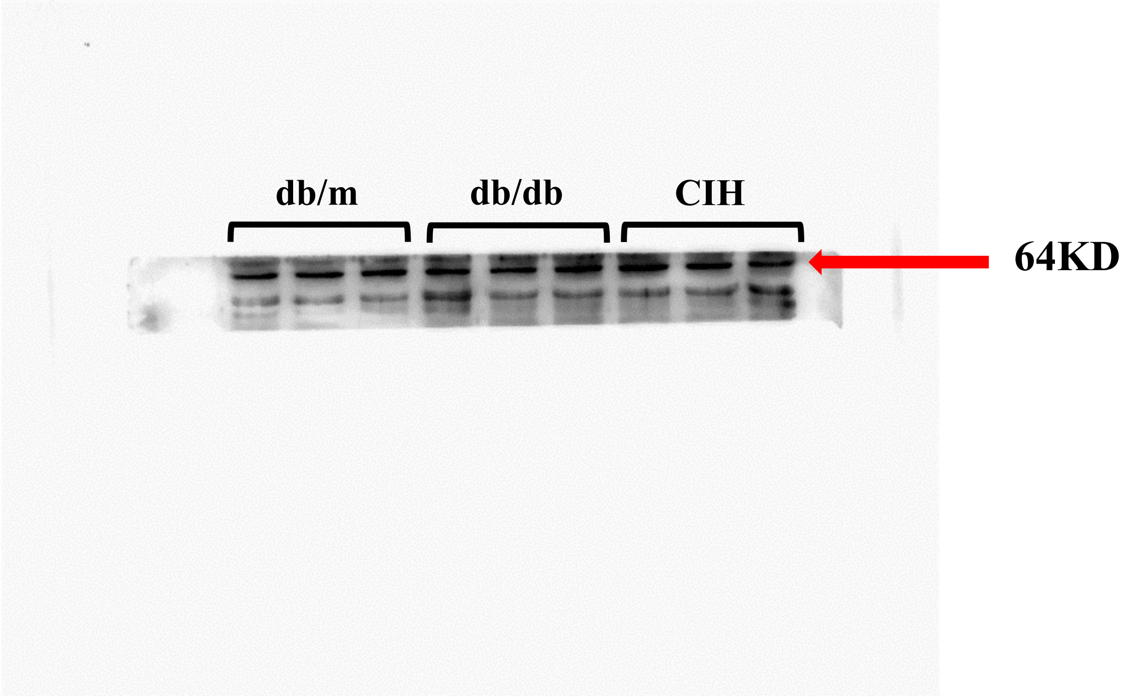

Nrf2

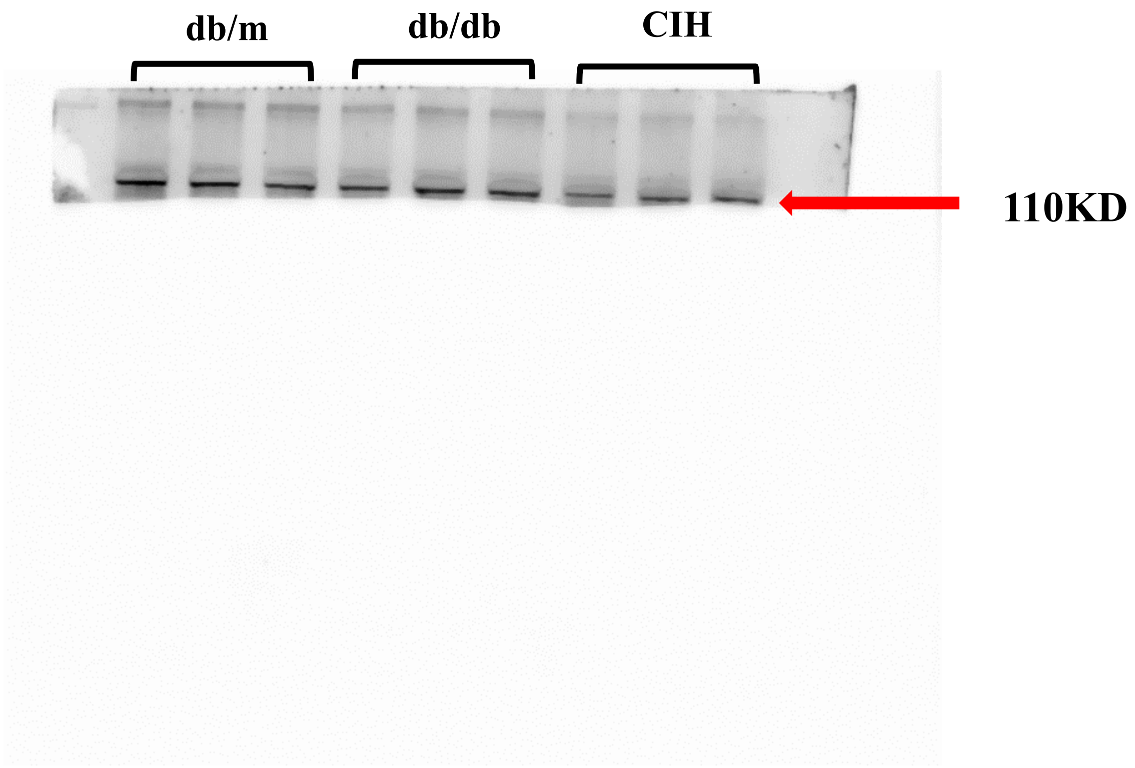

HO-1

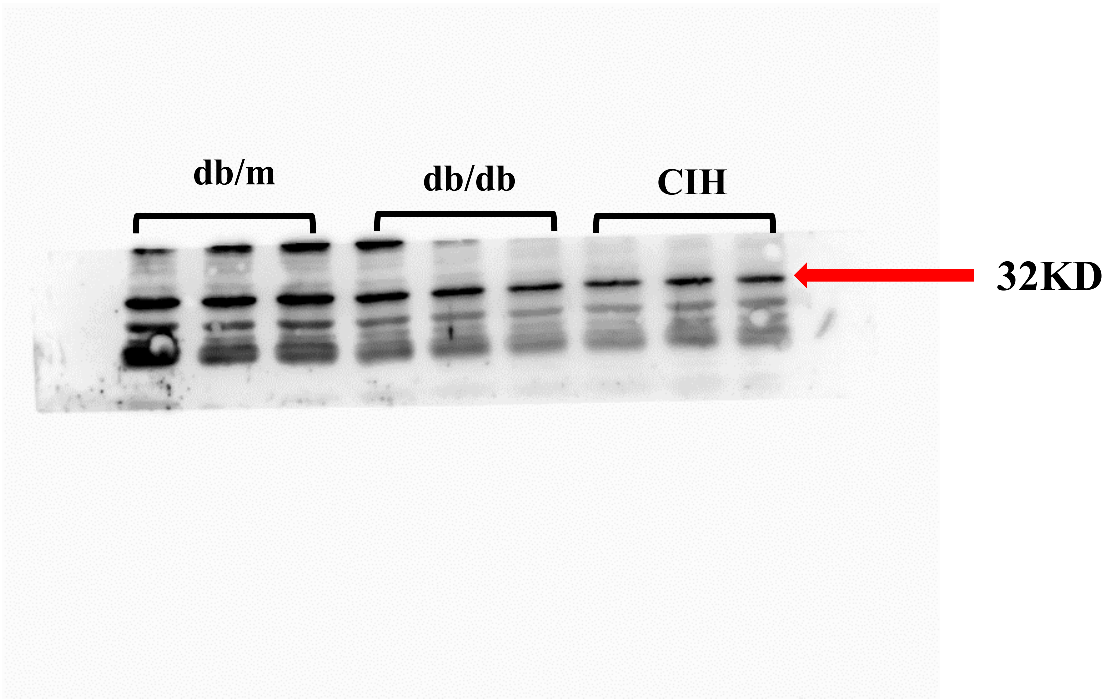

Tubulin

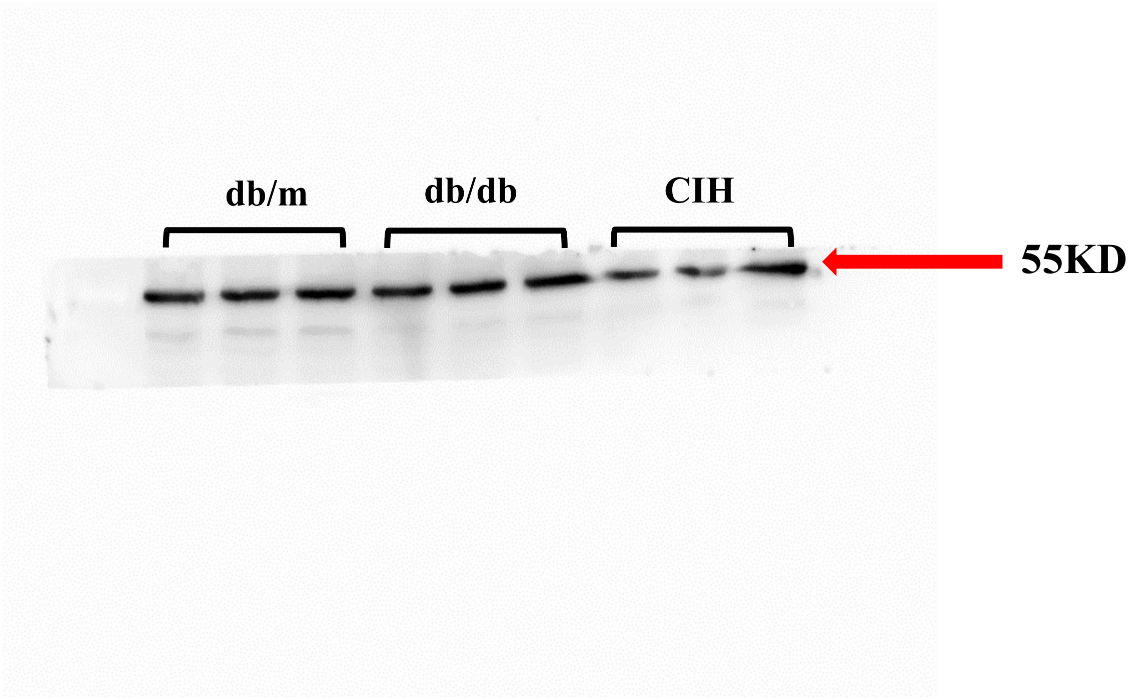

Nuclear-Nrf2

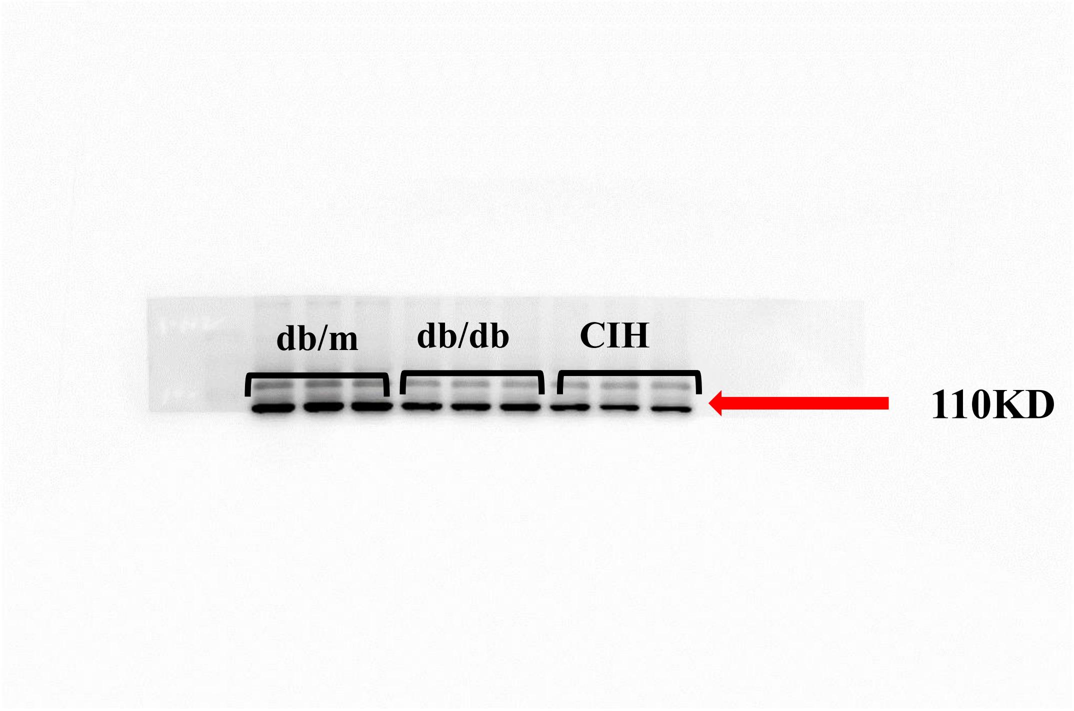

Lamin B1

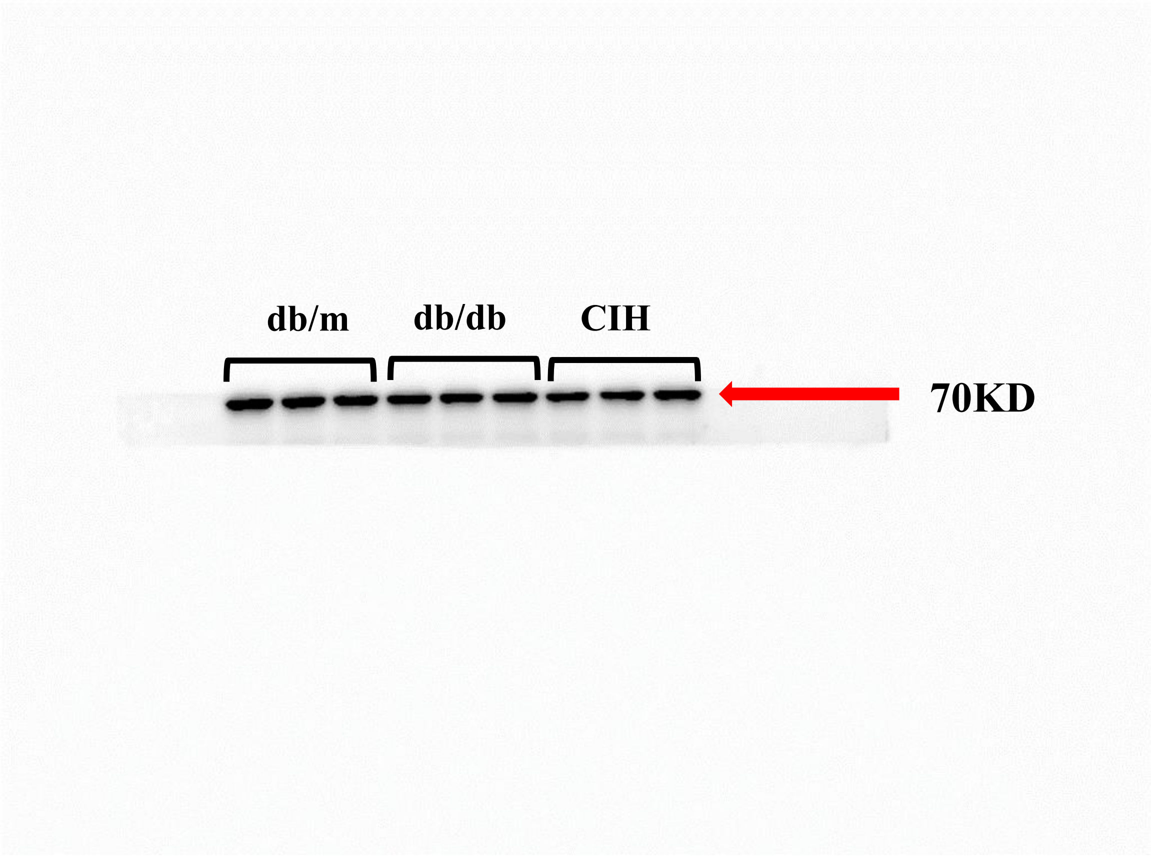

Figure 8: Representative blot images of LKB1, AMPK, and Nrf2 expression.

P-LKB1

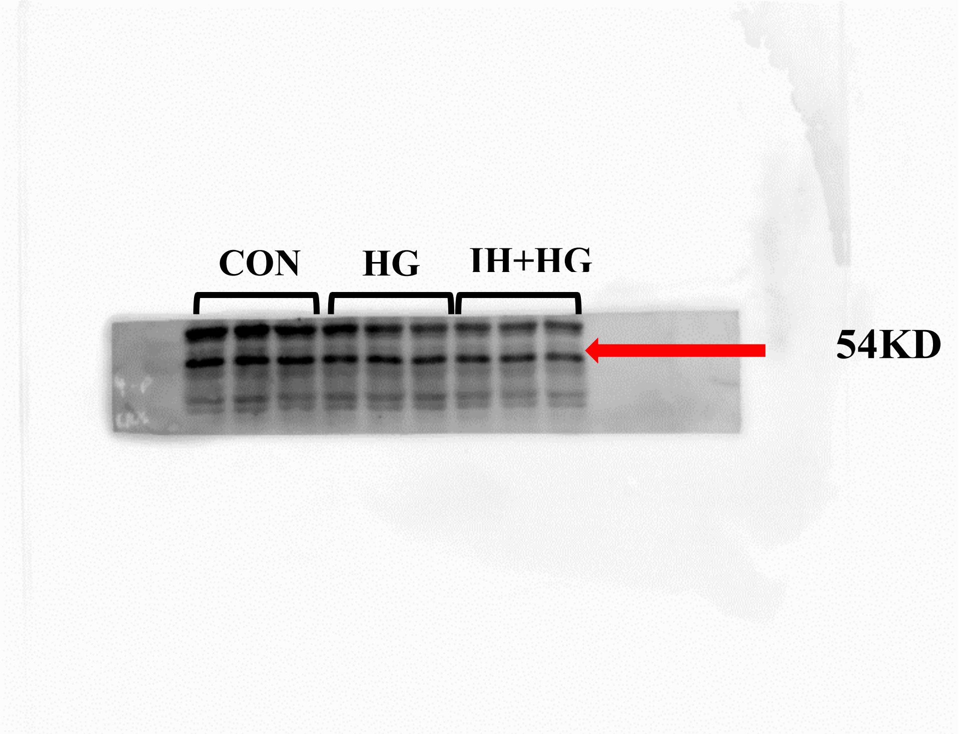

LKB1

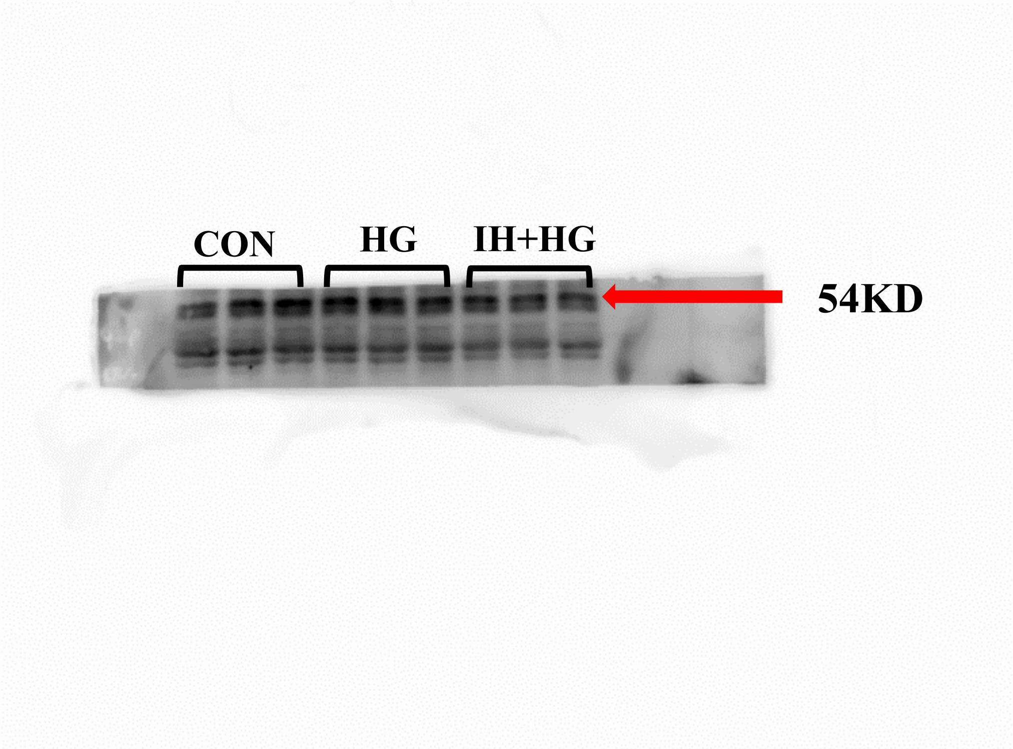

P-AMPK

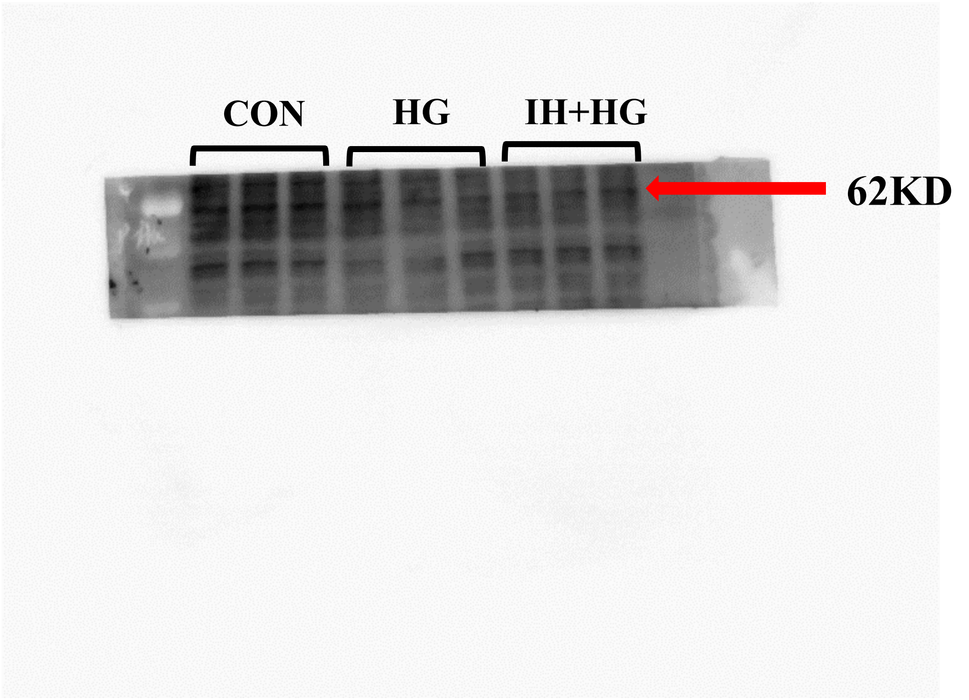

AMPK

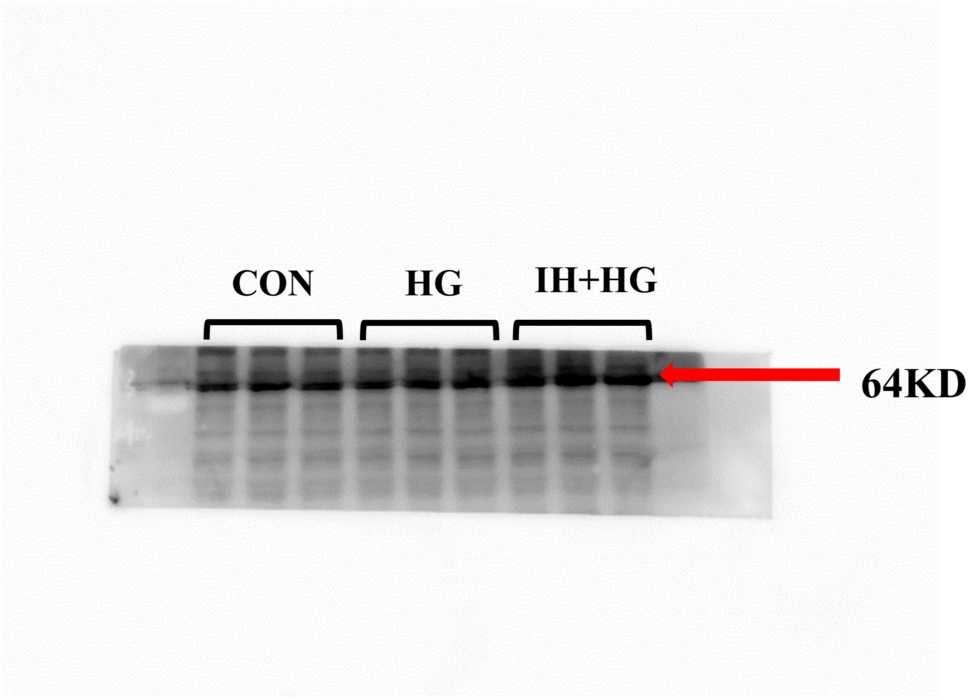

Nrf2

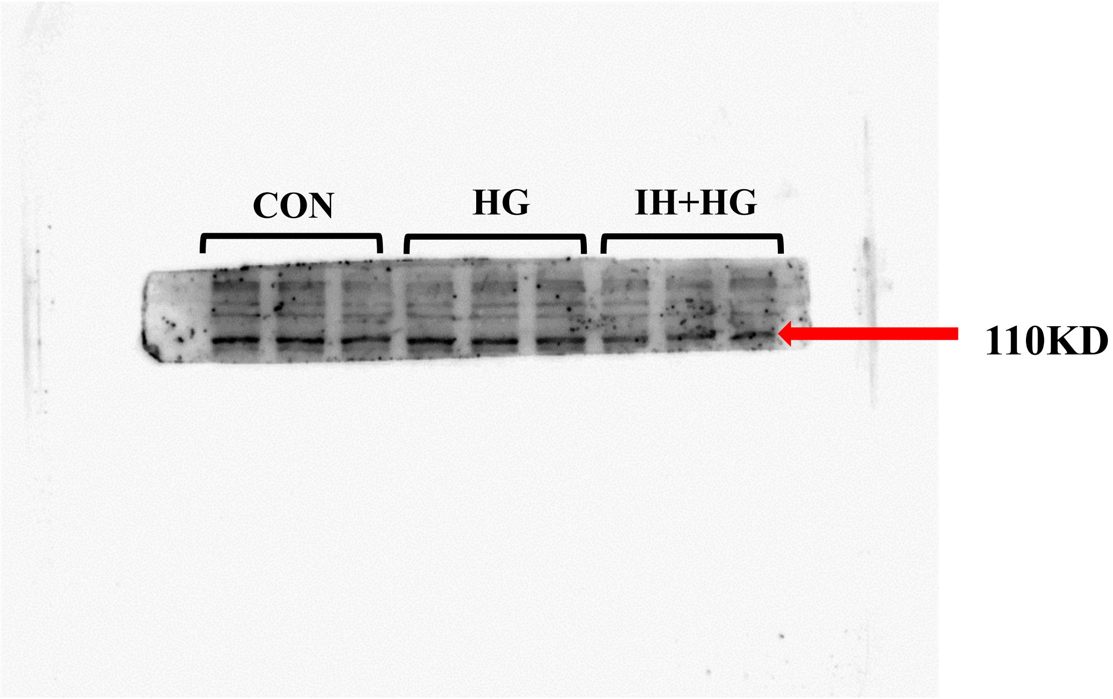

Tubulin

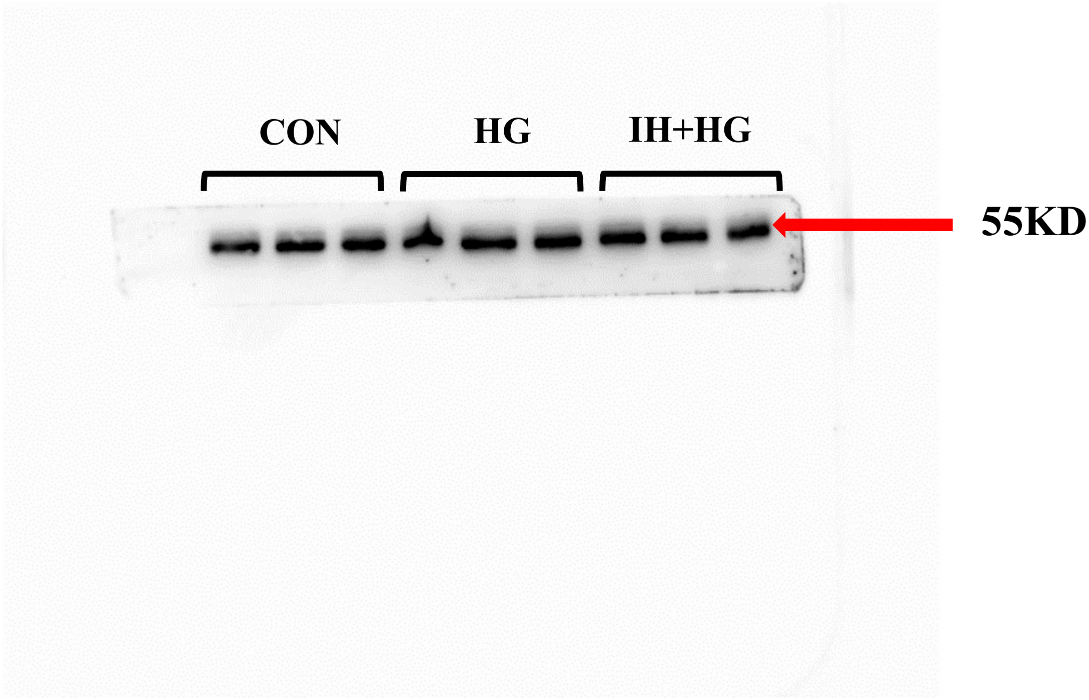

Figure 9: Representative blot images of AMPK and Nrf2 expression.

P-AMPK

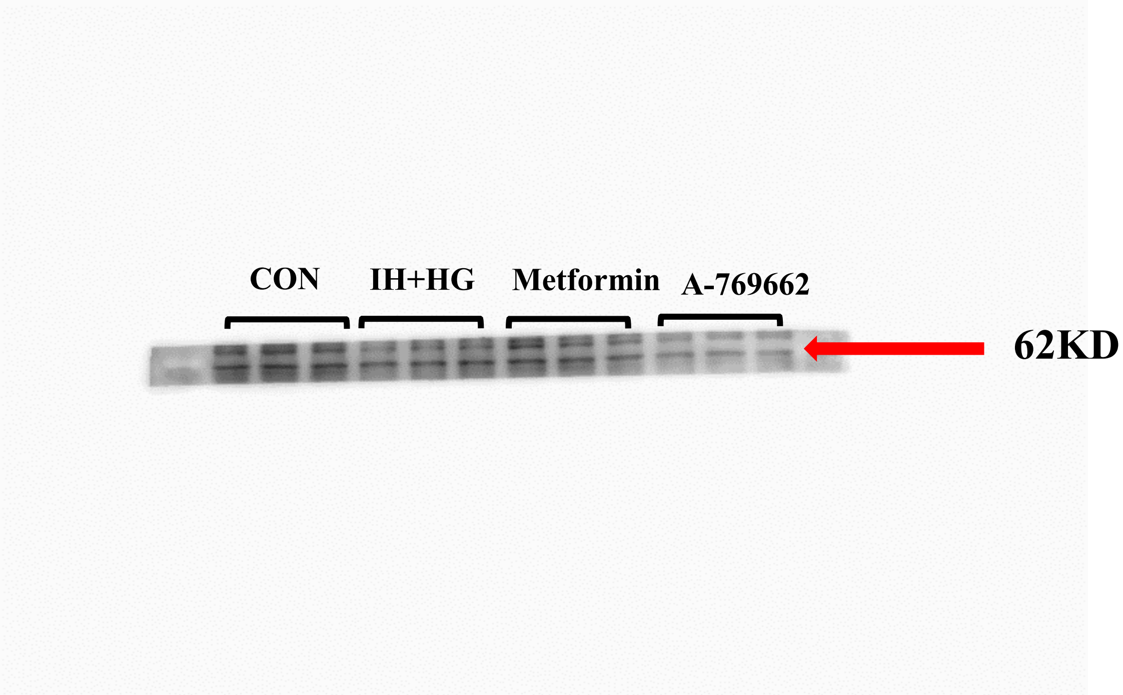

AMPK

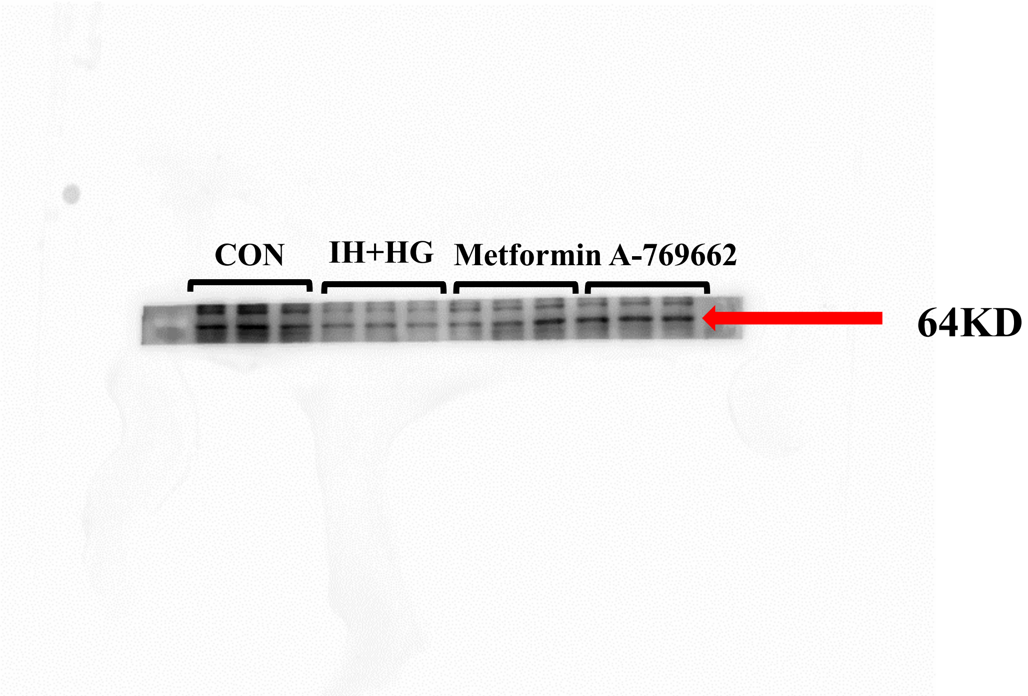

Nrf2

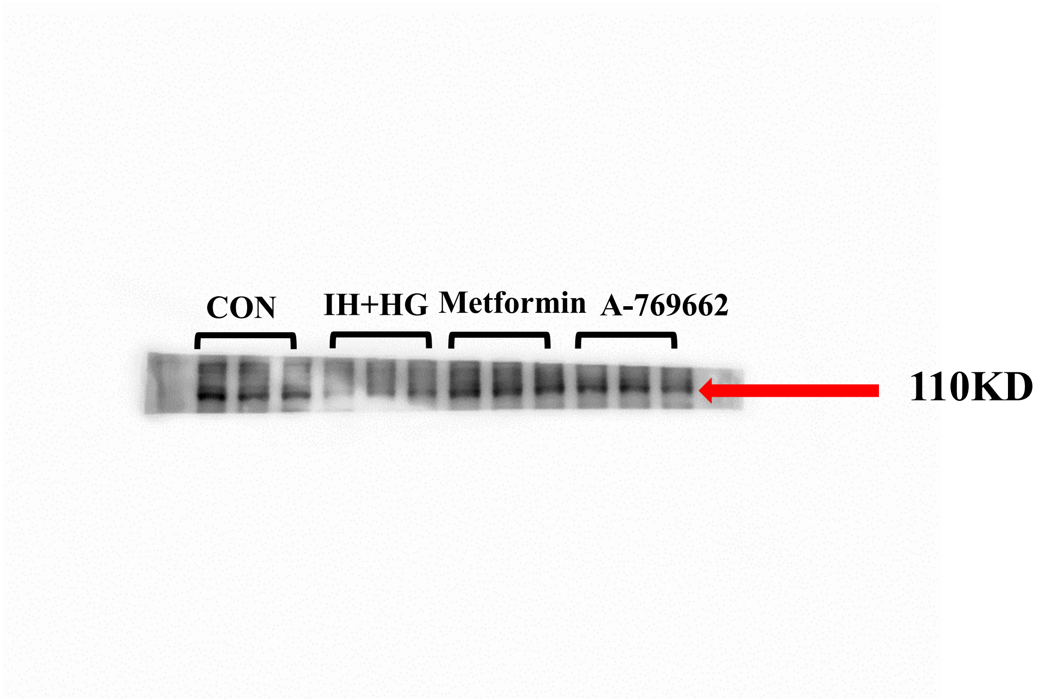

Tubulin

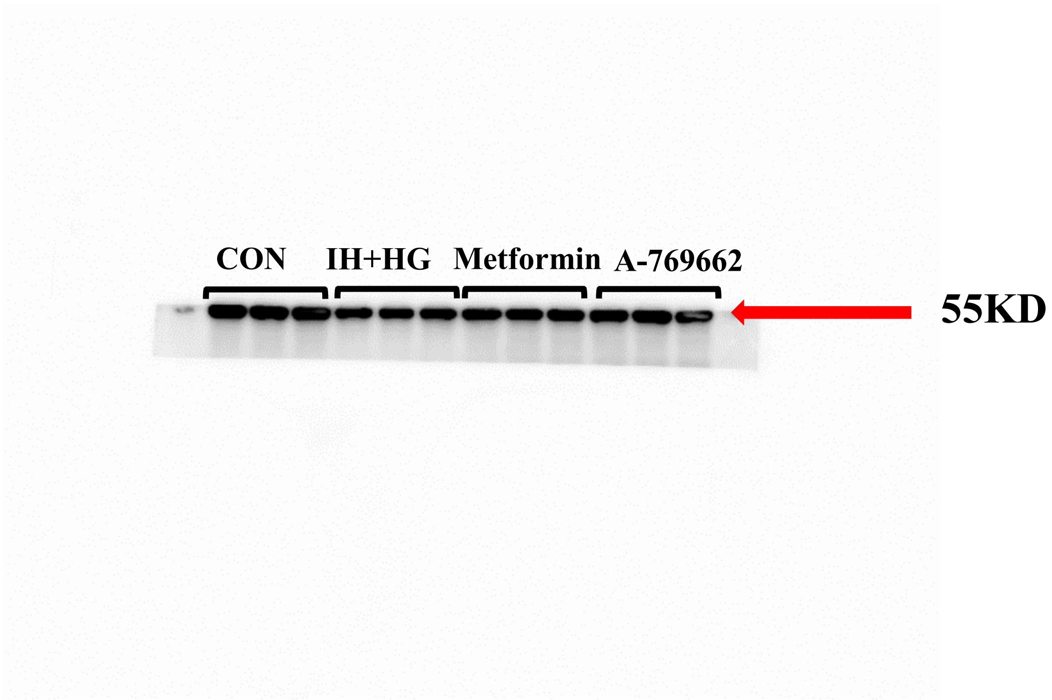

Supplement: S1 Raw images — (PDF) [file pone.0296792.s002.pdf]
